# The Nasal Endoscopic Features of Postnasal Drip: A Cross Sectional Study

**DOI:** 10.1055/s-0043-1767799

**Published:** 2023-05-29

**Authors:** Nur Eliana Tarmizi, Aneeza Wan Hamizan, Chong Sian Ng, Hardip Singh Gendeh, Lum Sai Guan, Farah Dayana Zahedi, Marina Mat Baki, Salina Husain

**Affiliations:** 1 Department of Otorhinolaryngology, Head and Neck Surgery, Universiti Kebangsaan Malaysia Medical Centre, Kuala Lumpur, Malaysia

**Keywords:** postnasal drip, PND, rhinitis, nasal mucosa, nasal endoscopy, laryngopharyngeal reflux

## Abstract

**Introduction** Patients with chronic rhinitis suffer from postnasal drip (PND) but this symptom is not well addressed. Nasal endoscopy may aid in identifying PND. Well described endoscopic features of PND are presence of secretions in the posterior nasal cavity, diffuse erythema, and hemorrhagic spots in the nasopharynx, but these have not been formally studied.

**Objectives** The present study aims to assess the association of nasal endoscopic features with PND among rhinitis patients. This will guide clinicians to interpret the nasal endoscopic findings appropriately.

**Methods** Adults (≥ 18 years old) with chronic rhinitis were consecutively recruited at an Otorhinolaryngology outpatient clinic in a tertiary referral center. The patients were grouped into either “Rhinitis with PND” or “Rhinitis only.” The endoscopic features of PND were scored as: Secretions in the posterior nasal cavity (yes/no), erythema in the nasopharynx (none, roof only, diffuse), hemorrhagic spots (yes/no), then were compared between groups.

**Results** There were 98 patients included (age 32.32 ± 11.33 years old, 61.2% female, 61.2% PND). Presence of secretions in the posterior nasal cavity was associated with PND (“Rhinitis with PND” versus “Rhinitis only,” 78.3 versus 55.3; *p* = 0.02; Odds ratio: 2.81; 95% confidence interval [CI]: 1.08–7.32). Diffuse erythema of the nasopharynx was more frequent in “rhinitis only” compared with those with PND (76.3 versus 53.3%; *p* = 0.02). Hemorrhagic spots were equally present in both groups (11.7 versus 18.4%; *p* = 0.35).

**Conclusion** Presence of secretions in the posterior nasal cavity may indicate bothersome PND among patients with rhinitis. Diffuse erythema of the nasopharynx and hemorrhagic spots are a nonspecific sign of inflammation.

## Introduction

Postnasal drip (PND) is the feeling of mucus secretion at the back of the throat. It was first defined as a sense of fullness deeply seated in the back of the nose with cough on intervals, with frequent hawking and spitting pellets of mucus. 1 Patients who complain of PND may describe a feeling of stagnant mucus at the back of the throat or may simply complain of nonspecific cough. It may also be confused with globus sensation of the throat. 2 This symptom is known to occur among patients with rhinitis (allergic or nonallergic), which is a common condition seen in the Otorhinolaryngology (ORL) clinic. Presence of PND will also require an assessment for chronic rhinosinusitis (CRS) and laryngopharyngeal reflux (LPR). 2 This is done by thorough history taking and physical examination which includes nasal endoscopy.

Nasal endoscopic findings commonly found based on clinical experiences are secretions in the posterior nasal cavity with redness or hemorrhagic spots in the nasopharynx. These features may be signs of PND, but this has not been formally studied. These clinical signs may be useful to support the symptom of PND and help to understand the mucosal changes associated with PND among patients with rhinitis.

The present study aims to assess the association of nasal endoscopic feature with PND among rhinitis patients. This will help clinicians to understand the mucosal changes seen in PND and interpret the nasal endoscopic signs appropriately.

## Materials and Methods

### Study Design

This was a cross-sectional study conducted at the ORL outpatient clinic at a tertiary referral center. Ethics approval and written informed consent was obtained from all patients (JEP-2019–760).

### Study Population

Adults (≥ 18 years old) newly referred for chronic rhinitis were consecutively recruited. Patients were included if they had at least two nasal symptoms (either nose block, runny nose, sneezing, or itchy nose) for at least 3 months. These rhinitis patients were grouped into either the “Rhinitis with PND” group or the “Rhinitis only” group based on the presence or absence of PND symptoms. Patients were excluded if there was history of nasal surgery, underlying systemic condition that affect the nasal mucosa such as autoimmune diseases, Wegener granulomatosis, cystic fibrosis, and systemic lupus erythematosus. Pregnant patients, recent URTI/nasal infections, patients who have chronic rhinosinusitis, and nasal tumors were also excluded.

All recruited patients underwent assessment for rhinitis and scored the severity of their nasal symptoms for the past 1 week using the visual analogue score (VAS) (0–100mm). These patients were defined to have allergic rhinitis if there were either a positive skin prick test or serum specific immunoglobulin E toward aeroallergens.

### Assessment for Postnasal Drip

All patients answered a series of self-reported yes/no questions regarding PND. The patients were asked if they had experienced symptoms of feeling drip in the throat, fullness in the nasopharynx, hawking, or intermittent cough and if they were bothersome. Postnasal drip was defined as presence of at least one PND symptom AND the patient indicated it as bothersome. Patients with these criteria were grouped as “rhinitis with PND” while those who did not fulfil these criteria were grouped as “rhinitis only.”

### Assessment for Laryngopharyngeal Reflux

All patients answered the reflux symptoms index (RSI) question independently 3 and underwent 70-degree angled endoscopy to assess the laryngeal findings. The recorded endoscopy video was scored using the reflux finding score (RFS) 4 by an ORL specialist. Laryngopharyngeal reflux is diagnosed if patients had an RSI > 13 and RFS > 7. 5

### Nasal Endoscopy Assessment

**Fig. 1:**
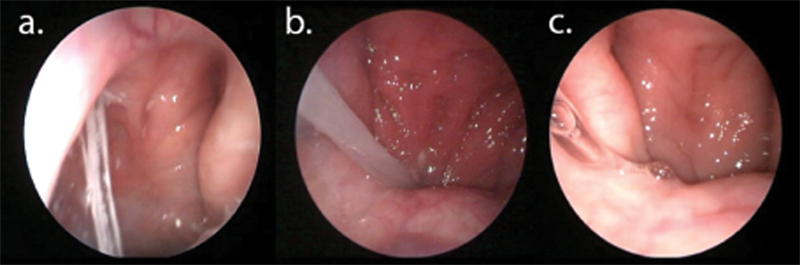
The reference image used by study blind assessors to grade the presence of secretions in the posterior nasal cavity. Presence of secretion is defined as secretions in the posterior nasal cavity or the choana either near septum (a) or eustachian tube which may be thick whitish (b) or clear secretions (c).

The nose of the patient was decongested with cophenylcaine forte spray 2 sprays each nostril for 15 minutes prior to nasal endoscopic assessment. The endoscopy was done using Hopkins II straight telescope 0 degree with a diameter of 4mm and a length of 18 cm. The nasal endoscopy was advanced until the nasopharynx to visualize the features of PND including secretions at the posterior nasal cavity, redness at the roof of the nasopharynx, and presence of hemorrhagic spots at the nasopharynx. The definition for these endoscopic features was made based on its endoscopic appearance. All nasal endoscopies were recorded, and the videos were anonymized. Two experienced rhinologists reviewed and graded the images guided by the reference images ( Figs. 1 2 3 ). The assessors were blinded to the PND status of the patient and their presenting symptoms. These two independent assessors reviewed a group of patients for interobserver reliability. For intraobserver reliability, the main assessor also rescored the same group 8 weeks later.

**Fig. 2:**
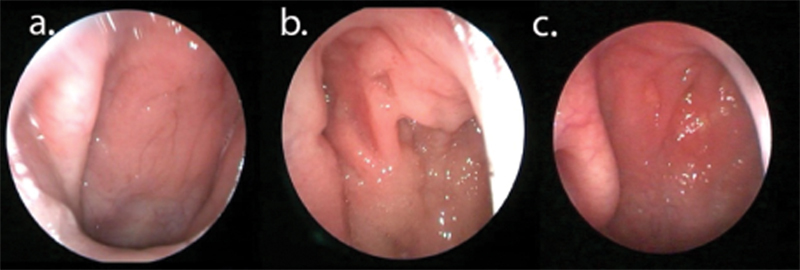
The reference image used by study blind assessors to grade the redness roof of nasopharynx. Redness of nasopharynx is defined as erythematous mucosa over nasopharynx. It has been graded as Grade 0 which is no redness of nasopharynx (a). Grade 1 (roof only), defined as redness involving only roof of nasopharynx, (roof defined as the level of above the upper 1/3 ^rd^ edge of the torus tubarius (b). Grade 2 (diffuse), defined as redness extending beyond the roof of the nasopharynx (c).

**Fig. 3:**
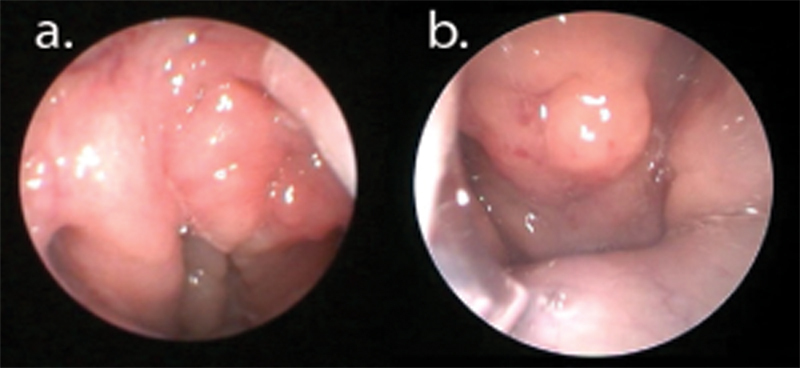
The reference image used by study blind assessors to grade the presence of hemorrhagic spots in the nasopharynx. It is defined as multiple reddish spots in the nasopharynx. It will be graded either as absent (a) or present (b).

### Secretions at the Posterior Nasal Cavity

Secretion at the posterior the nasal cavity is defined as the presence of thick whitish or clear mucus in the posterior nasal cavity tracking down from the choana ( Fig. 1 ). The rater will grade either yes or no depending on the presence of secretions at the posterior nasal cavity.

### Redness in the Roof of the Nasopharynx

A grading system to assess redness of the nasopharynx was developed after reviewing the images of the nasopharynx and consensus was reached between three experienced rhinologists (Hamizan A., Zahedi F., Husain S.). Redness of the nasopharynx is defined as erythematous mucosa over the nasopharynx. It has been graded as Grade 0: no redness of the nasopharynx. Grade 1 (roof only): redness involving the only roof of the nasopharynx, (roof is defined as the level above the upper 1/3 ^rd^ edge of the torus tubarius). Grade 2 (diffuse): redness extending beyond the roof of the nasopharynx ( Fig. 2 ).

### Presence of Hemorrhagic Spots

Hemorrhagic spots are defined as multiple reddish spots at the nasopharynx. It was documented either as absent or present ( Fig. 3 ).

### Statistical Analysis

Statistical analysis was done using IBM SPSS Statistics for Windows version 26 (IBM Corp., Armonk, NY, USA). Descriptive data for the proportions and percentages were calculated for categorical variables. Categorical data was analyzed using chi-squared tests, ordinal data with the Kendall tau B test, and continuous data with the Student *t* -tests. Association between nasal endoscopic features and rhinitis with or without PND groups was analyzed using the chi-squared test and Kendall tau b. Binary logistic regression was used to assess probability of PND from nasal endoscopic features. The interrater reliability for endoscopic scores was analyzed by Cohen kappa. A *p* -value < 0.05 was considered statistically significant. The sensitivity, specificity, positive predictive value (PPV), negative predictive value (NPV), likelihood ratio positive (LR + ) and likelihood ratio negative (LR-) were calculated for endoscopic features which proved to have an association with PND.

## Results

There were 98 participants, of which 61.2% were female with a mean age of 32.32 ± 11.33 years old. There were 61.2% with PND and the most common PND symptoms reported were feeling drip (65.3%), followed by fullness of the nasopharynx (57.1%), hawking (51%), and intermittent cough (42.9%). The patients were grouped into either the “rhinitis with PND” group or the “rhinitis only” group. The baseline characteristics between these groups are compared in Table 1 . The rhinitis with PND group has more participants with moderate to severe AR (85 versus 57.9%, *p* < 0.01) and more severe VAS (65.82 ± 23.94 versus 44.58 ± 27.0; *p* < 0.01). There were no significant differences between these two groups in terms of age, gender, duration, asthma, and usage of intranasal corticosteroids.

**Table 1:** Baseline characteristics between PND and rhinitis only group

	Rhinitis with PND	Rhinitis only	*p-value*
*n* (%)	60 (61.2%)	38 (38.8%)	−
Age (years old) (mean ± SD)	30.97 ± 9.55	34.44 ± 13.53	0.14
Gender (%female)	63.3	57.9	0.59
Allergic rhinitis %	88.3	73.7	0.06
Moderate to severe AR %	85	57.9	<0.01
Persistent rhinitis %	60	50	0.33
Duration of rhinitis			
*< 2 years*	13.3	23.7	0.56
*2–4 years*	23.3	18.4	
*5–10 years*	20	23.7	
*>10 years*	43.3	34.2	
VAS overall rhinitis (mean ± SD)	65.82 ± 23.94	44.58 ± 27.0	<0.01
Asthma %	33.3	21.1	0.19
Use of intranasal corticosteroids %	6.7	7.9	0.82

Abbreviations: AR, Allergic rhinitis; PND, postnasal drip; VAS, Visual analoque scale.

### Assessment of LPR among Rhinitis Patients

Among all participants, there were 10 patients (10.2%) with LPR (RSI >13 and RFS >7). There was no difference in LPR between these two groups (13.3 versus 5.3%; *p* = 0.20). The “rhinitis with PND” group had a higher RSI score compared with the “rhinitis only” group (14.70 ± 11.99 versus 6.58 ± 6.14; *p* < 0.01) but with similar RFS scores (5.0 ± 4.23 versus 4.82 ± 4.34; *p* = 0.84).

### Nasal Endoscopic Features of Rhinitis

A total of 69.4% of the participants had secretions in the posterior nasal cavity. Among the participants, 62.2% had diffuse redness of the nasopharynx, 29.6% had only redness at the roof, and 8.2% had no redness. A total of 14.3% of participants had hemorrhagic spots in the nasopharynx.

### Association Between Nasal Endoscopic Features and PND

Secretions in the posterior nasal cavity were higher among the “rhinitis with PND” group compared with “rhinitis only” (78.3 versus 55.3%; *p* = 0.02). Redness of the nasopharynx between “rhinitis with PND” compared with “rhinitis only” are as follows: none (10 versus 5.3%), roof only (36.7 versus 18.4%), and diffuse redness (53.3 versus 76.3%; *p* = 0.02). The presence of hemorrhagic spots were similar between “rhinitis with PND” compared with “rhinitis only” (11.7 versus 18.4; *p* = 0.35). There is also no difference between groups for granular posterior pharyngeal wall (63.3 versus 71.1; *p* = 0.43). Logistic regression analysis indicated that PND was more likely when there are secretions in the posterior nasal cavity (Odds ratio [OR]: 2.81; 95% confidence interval [CI]: 1.08–7.32) ( Table 2 ). The presence of secretions is 78.3% sensitive, 44.7% specific, 69.1% positive predictive value (PPV) and 56.6% negative predictive value (NPV), with a likelihood ratio positive 1.41 and likelihood ratio negative 0.48 to predict PND among rhinitis patients.

**Table 2:** Binary logistic regression model to predict postnasal drip using nasal endoscopic features among patients with chronic rhinitis

Variables	OR (95%CI)	Sig
Constant	3.95	0.24
***Nasal endoscopic features (Base: score of 0)***		
Secretions in the nasal cavity	2.81(1.08–7.32)	**0.04**
Redness of nasopharynx		0.13
*Roof only*	1.27 (0.20–8.26)	0.44
*Diffuse redness*	0.44 (0.08–2.52)	0.36
Hemorrhagic spot	0.45 (0.13–1.57)	0.21
Chi squared	0.04	
-2LL	117.73	
Negelkerke R2	0.17	
Cox and Snell R2	0.13	
Hosmer & Lemeshow test	*p* = 0.08	
Classification accuracy	66.3%	

Abbreviations: CI, confidence interval; OR, odds ratio.

#### Inter-rater and Intrarater Reliability

The inter-rater and intrarater reliability for the grading of redness of the nasopharynx was 0.84 (95%CI: 0.68–0.99; *p* < 0.01) and 0.80 (95%CI: 0.64–0.96; *p* < 0.01), respectively.

## Discussion

Nasal endoscopy is an easily available tool to an otorhinolaryngologist and may aid in the management of PND. In this rhinitis population, the majority of rhinitis patients who complain of PND have detectable secretions in the posterior nasal cavity and are 2.8 times more likely to have PND. This may be used as an objective finding to support the presence of PND. Another study also reported that 9 out of 10 rhinosinusitis patients with purulent secretions do complain of PND. However, it is poorly specific where more than half of rhinitis patients without PND have this finding. Therefore, PND should not be determined based on nasal endoscopic finding alone. The mechanism of PND still remains controversial and should not be oversimplified as mechanical irritation from the nasal secretions. In an experiment, Rimmer et al. demonstrated that rhinitis patients with PND were not able to perceive the sensation of simulated mucus compared with healthy controls. 6 This may be because patients with PND tend to have viscid secretions containing inflammatory cells and neuropeptides that cause inflammation and dysesthesia of the nasopharynx. 6 7 This may also explain the poor specificity of posterior nasal cavity secretions to predict PND.

Redness of the nasopharynx was found to be higher among patients with rhinitis only compared with the PND group. This suggests that the erythema in the nasopharynx is due to inflammation associated with rhinitis itself 8 rather than a specific sign of PND. Inflammation of the nasopharynx or nasopharyngitis has also been described as a sign of LPR, 9 10 but based on this current finding, nasopharyngeal inflammation may also be due to rhinitis. The presence of hemorrhagic spots and the granular posterior pharyngeal wall was equally present among rhinitis patients with or without PND. Based on current evidence, redness and hemorrhagic spots are likely not related to PND but are nonspecific signs of inflammation. Histological examination of the nasopharynx of patients with primary PND was also reported to be similar to chronic rhinitis. 11 Therefore, erythema of the nasopharynx is not a specific sign of PND but indicates inflammation which may be due to rhinitis itself, LPR, or even breathing in dry air. 12 13

In this rhinitis population, more than half complained of bothersome PND. This is comparable to Jaruvongvanich et al. 14 which reported that 56.3% of patients with allergic rhinitis had at least moderately severe PND. In the present study, patients with PND had more severe overall rhinitis symptoms compared with those without PND (VAS: 65.82 ± 23.94 versus 44.58 ± 27.0; *p* < 0.01). The severity of nasal symptoms may be assessed using patient reported outcomes such as the overall VAS score, total nasal symptoms score (TNSS) or by the rhinitis quality of life score. 15 Del Cuvillo et al. 16 reported that a VAS < 40 mm indicated mild symptoms, 40 to 70 mm indicated moderate nasal symptoms while a score of more than 70 mm indicates severe allergic rhinitis. Therefore, patients with PND tended to have moderate to severe AR. This suggests that treating the rhinitis first will reduce PND. The VAS was used in the present study as it is a simple method to assess the severity of nasal symptoms. It correlates well ( *p* < 0.01) with the total nasal symptom score (R = 0.59) as well as the rhinitis quality of life questionnaire (R = 0.68). 16 A reduction in VAS also correlated well with reduced TNSS and improved quality of life. 17

There is a tendency to prescribe proton pump inhibitors to treat LPR among the rhinitis population. This is because symptoms tend to overlap and studies have shown association between LPR and allergic rhinitis. 18 Furthermore, the RSI and RFS scores for allergic rhinitis patients tend to mimic the scores for LPR, making it very difficult to distinguish between the two pathologies. 19 In the present study, rhinitis patients with PND tend to have secretions in the posterior nasal cavity and this may be used to support rhinitis as the underlying cause of PND. This also implies that patients who suffer from PND but lack secretions in the posterior nasal cavity may have underlying LPR and require further assessment.

The redness of the nasopharynx and granular posterior pharyngeal wall that was assessed appeared to have good test characteristics. Both inter-and intraobserver Cohen Kappa and ICCs were good and it is likely that the use of reference images and predefining the appearance of redness and granularity of the posterior pharyngeal wall contributed to this finding. The limitation of the present study is the lack of a Hypopharyngeal-Esophageal Multichannel Intraluminal Impedance with dual pH probe (HEMII-pH) testing which is considered as gold standard to exclude LPR in this study population. However, based on RSI and RFS, LPR was only present in 10.2% in the whole study population and the proportion of LPR was not significantly different between groups (13 versus 5%; *p* = 0.2). Future studies investigating the relationship of postnasal drip with LPR confirmed by HEMII-PH study will be useful. 20 Another limitation is that diffuse redness of the nasopharynx was not further graded according to the severity (mild, moderate, or severe) but this is difficult to evaluate subjectively. The statistical analyses method to assess these endoscopic features based on previous studies to objectively assess its usefulness to predict PND 21 but the results may differ in other patient populations. Redness of the nasopharynx and hemorrhagic spots without secretions may be more suggestive of LPR, but this requires further study which separates LPR, rhinitis only, and healthy controls.

Taken together, these findings suggests that secretions in the posterior nasal cavity is a sign of PND in rhinitis patients and should alert ORL doctors to treat the associated PND. Redness of the nasopharynx is a nonspecific sign of inflammation from various underlying etiologies. Therefore, rhinitis patients who complain of PND and positive findings of secretions in the posterior nasal cavity should receive nasal douching together with routine intranasal steroids. 7 14 Patients who do not complain of rhinorrhea and do not have secretions in the posterior nasal cavity but have PND may be further evaluated for other etiologies.

## Conclusion

Presence of secretions in the posterior nasal cavity may indicate bothersome PND among patients with rhinitis. Diffuse erythema of the nasopharynx and hemorrhagic spots are nonspecific signs of inflammation but are not signs of PND. Careful evaluation of symptoms together with nasal endoscopic findings may help guide further management.
